# Therapeutic Results and Survival of Patients with Myelofibrosis Treated with Ruxolitinib—A Real-Life Longitudinal Study

**DOI:** 10.3390/cancers15205085

**Published:** 2023-10-20

**Authors:** Vera Stoeva, Georgi Mihaylov, Konstantin Mitov, Guenka Petrova, Konstantin Tachkov

**Affiliations:** 1 Specialized Hospital for Active Treatment of Hematological Diseases, 1000 Sofia, Bulgaria; 2 Faculty of Pharmacy, Medical University of Sofia, 1000 Sofia, Bulgariaktashkov@pharmfac.mu-sofia.bg (K.T.)

**Keywords:** myelofibrosis, ruxolitinib, therapeutic results, survival analysis

## Abstract

**Simple Summary:**

Myelofibrosis is a rare blood cancer, the onset of which usually occurs at an advanced age. Real-life studies on the influence of ruxolitinib on therapeutic results and patient survival are scarce for the disease both worldwide and in Bulgaria, provoking our interest towards this topic. The aim of this study was to analyze the therapeutic results and survival of patients with myelofibrosis treated with ruxolitinib in comparison to standard therapy.

**Abstract:**

The aim of this study was to analyze the therapeutic results and survival of patients with myelofibrosis treated with ruxolitinib in comparison with a group on standard therapy. It is a cross-sectional, retrospective, non-interventional, real-life study that was performed between January 2000 and February 2023. Patients treated between 2000 and 2016, before the introduction of ruxolitinib, constituted the control group (*n* = 45), while those treated after May 2016, after ruxolitinib inclusion, constituted the active group (*n* = 66). Demographic characteristics, clinical indicators, the severity of the disease, and survival were explored using Kaplan–Meier survival analyses. Spearman’s correlation, linear regression, and other statistical analyses were performed. According to the Kaplan–Meier analysis, there was a 75.33% reduction in the fatality risk in the sample. On a general-population level, the fatality risk in the group treated with ruxolitinib varied between 7.9% and 77.18% compared to that of the risk in the control group. There was a decrease in blood parameters (leukocytes, hemoglobin, and platelets) and spleen size. During the first six months, the spleen size of the patients on ruxolitinib decreased by 6%, and during the second six months, it decreased by another 9%. This study shows that patients in a real-life clinical setting treated with ruxolitinib exhibited improved clinical signs of the disease, had a lower symptom severity, and survived longer than patients on standard therapy before ruxolitinib’s entrance into the national market. The improvements correlate with those reported in randomized clinical trials.

## 1. Introduction

Rare diseases are defined as having a prevalence of 5 in 10,000 people in Europe. Myelofibrosis (MF) is a rare disease with a prevalence of 1 to 1.5 per 100,000 individuals worldwide. It is understudied; therefore, studies about the results of its therapy are scarce. MF is a blood cancer with an onset at an advanced age [1]. It is a rare, chronic disease related to abnormal blood cell development that can lead to fatigue, shortness of breath, abdominal discomfort, pain under the ribs, loss of appetite, muscle and bone pain, itching, and night sweats [2]. Together with an enlarged spleen, these symptoms form the basis for an MF diagnosis [3].

Previously, the standard therapy for MF included blood transfusions, medicines, radiation therapy, spleen removal surgery, and stem cell transplantation, which was considered the only curative therapy [4]. Until recently, the primary goal of treatment was symptom relief, and conventional therapies were unable to significantly affect the course of the disease.

The contemporary management of MF in adults worldwide is based on two medicines—ruxolitinib and fedratinib [5,6,7]. Both products are from the Janus kinase inhibitor (JAKi) group, with ruxolitinib inhibiting JAK1 and JAK2 and fedratinib selectively inhibiting JAK2 [8]. JAKs are important triggers in intracellular pathways responsible for the release of the cytokines and growth factors necessary for hemopoiesis. Both JAK inhibitors are authorized for sale in Europe but are not available in all countries.

The efficacy and safety of ruxolitinib were investigated in two pivotal phase-3 randomized clinical trials (RCTs), COMFORT I and II [9,10]. The effectiveness of ruxolitinib was measured using two main clinical indicators—a reduction in the spleen and an improvement in the symptoms—for which, ruxolitinib was superior to both a placebo and the best available therapy.

The overall survival (OS) is one of the key long-term therapeutic outcomes that was measured in the RCTs and is used in everyday clinical practices. The influence of ruxolitinib on the overall survival was also investigated in clinical trials [11]. However, real-life studies investigating the influence of ruxolitinib on the therapeutic results and on the survival of patients with MF are scare, both worldwide and in Bulgaria, provoking our interest towards this topic [12].

The aim of this study was to analyze the therapeutic results and survival of patients with MF in Bulgaria by comparing patients treated with ruxolitinib after its inclusion into MF treatment regimes versus patients treated with the standard therapy prior to ruxolitinib’s inclusion.

## 2. Materials and Methods

### 2.1. Design of the Study

This study was a cross-sectional, retrospective, non-interventional, real-life study that was performed at the Specialized Hospital for Active Treatment of Hematological Diseases (SHATHD) in Sofia, Bulgaria, during the period of January 2000–February 2023 and included all patients with MF in the outpatient department of the hospital.

Patients treated between 2000 and 2016 constituted the control group (*n* = 45) and those treated after May 2016, when ruxolitinib was introduced into the market, constituted the active group (*n* = 66). The information for the patients was retrospectively collected by physicians from the available documentation at the hospital. The inclusion criteria were a confirmed MF diagnosis, treatment at SHATHD, and the use of standard therapy or ruxolitinib. The exclusion criterion was non-MF patients.

Ruxolitinib is prescribed according to the recommendations described in the summary of product characteristics (SPC) for the treatment of disease-related splenomegaly or symptoms in patients with primary MF, post-polycythemia vera MF, or post-essential MF thrombocythemia [13]. A specialized hematology committee makes the decision on whether the patient can be treated with ruxolitinib. When the patient starts treatment, they needs to visit the outpatient department every month. A blood test is conducted and, if necessary, the doctor can change the drug dosage. Every six months, the response is evaluated. Symptom severity is assessed with a specialized tool (MPN SAF TSS), while disease progression is evaluated through spleen size measurements. Ultrasound and CT scans are used for accurate spleen size measurements. The treatment continues until there is disease progression or an intolerance to the treatment develops. If a treatment change is indicated, it is discussed with the committee [14]. For the control group, such a protocol was not available.

For both groups, physicians collected demographic information, the type of MF (primary or secondary), pharmacotherapy and dosage regime, date of diagnosis, age at diagnosis, JAK2 status, need for hemotransfusions, mortality, and date of death.

For the patients in the ruxolitinib group, additional information was available regarding relevant changes in clinical outcomes, as well as for MF burden over the follow-ups after six months and one year of therapy. The burden of the symptoms is routinely evaluated with the Myeloproliferative Neoplasm (MPN) Symptom Assessment Form Total Symptom Score (MPN-SAF TSS) questionnaire. The questionnaire uses a 10-point scoring system, assessing fatigue, concentration, early satiety, inactivity, night sweats, itching, bone pain, abdominal discomfort, weight loss, and fever. A decreasing MPN SAF score indicates an improvement of symptoms.

This study was approved by the Specialized Hospital for Active Treatment of Hematological Diseases (SHATHD) in Sofia (Order 3-31/2022) and by the ethical committee of the SHATHD (decision 3-92#1/19.08.2022). All patients signed an informed consent form.

### 2.2. Survival and Statistical Analysis

Kaplan–Meier survival curves were first built in Excel [15] and the input data were then analyzed using MedCalc ver.13. For each subgroup (control on standard therapy and active group on ruxolitinib), the patients were coded with a serial number to protect their identity during the observation period. The mortality status of the patients at the end of period (dead or alive) was noted for both groups. A survival analysis was conducted for both groups, as well as individually according to gender and JAK status. The data for 3 patients needed to be censored.

A separate subgroup analysis was performed with the collected information in the active group to explore the correlation between the survival and changes in the clinical indicators. The Friedman method was used because, within the sample, the data for these values were dependent and not normally distributed.

A variety of statistical analyses were performed to test the significance of the changes in clinical indicators and their correlation with symptom severity and survival. The Friedman test was used for dependent variables. Spearman correlation analysis was performed to explore correlation between changes in the clinical indicators and survival.

Linear regression analysis was performed to analyze the prognostic value of decreases in spleen size and MPN-SAF TSS for survival.

## 3. Results

### 3.1. Demographic Characteristics and Dosage Regimes of the Observed Groups

The demographic characteristics of the observed patients are presented on Table 1. Although the average age of the patients in the control group is higher, disease onset usually occurred at advanced ages in both groups.

In both groups, the majority of patients were male, while primary MF was observed in half of the control group. Half of the patients in both groups were positive for the JAK2 mutation, either as a homo- or heterozygous mutation. We should mention that the periods of observation were almost equal for both groups.

The average length of the disease was shorter in the control group; however, one patient was an outlier with a 10-year survival. Transfusion dependence was observed in 11% of the patients in the active group, which is 5 times lower than the incidence in the control group.

Medicinal therapy in the control group was very scarce, with 21 patients treated with hydroxycarbamide, 5 with corticosteroids, 1 with interferon, and 3 with erythropoietin. During the period of observation, other treatment options for the control group were available such as interferon, lenalidomide, and thalidomide but they were not applied in the hospital.

One of the patients from the control group was later transferred onto ruxolitinib therapy when it was introduced into reimbursement practice in 2016.

In the active group, all patients were treated with ruxolitinib. The initial dose was defined by the treating physician. At the beginning of the therapy, 62% were assigned to the high dosage regimen of 20 mg ruxolitinib twice daily (BID), 21% were put on 15 mg BID, 11% on 10 mg BID, and 6% on 5 mg BID.

After six months, the dosage was reduced for 45% of the patients, and after one year of therapy, it was reduced again for a further 35% of the patients. There was no increase in the prescribed dosage regimes.

### 3.2. Therapeutic Results Analysis

For both groups of patients, we analyzed the long-term results of therapy based on the data from patient’s dossiers. Of particular interest was mortality, with all fatality cases occurring within the observed period being recorded.

The intermediate results for the changes in spleen volume, clinical tests, and symptoms severity were available only for the group on ruxolitinib and therefore were analyzed as separate subgroup analysis.

#### 3.2.1. Survival Analysis

##### Survival in the Control Group

**Figure 1:**
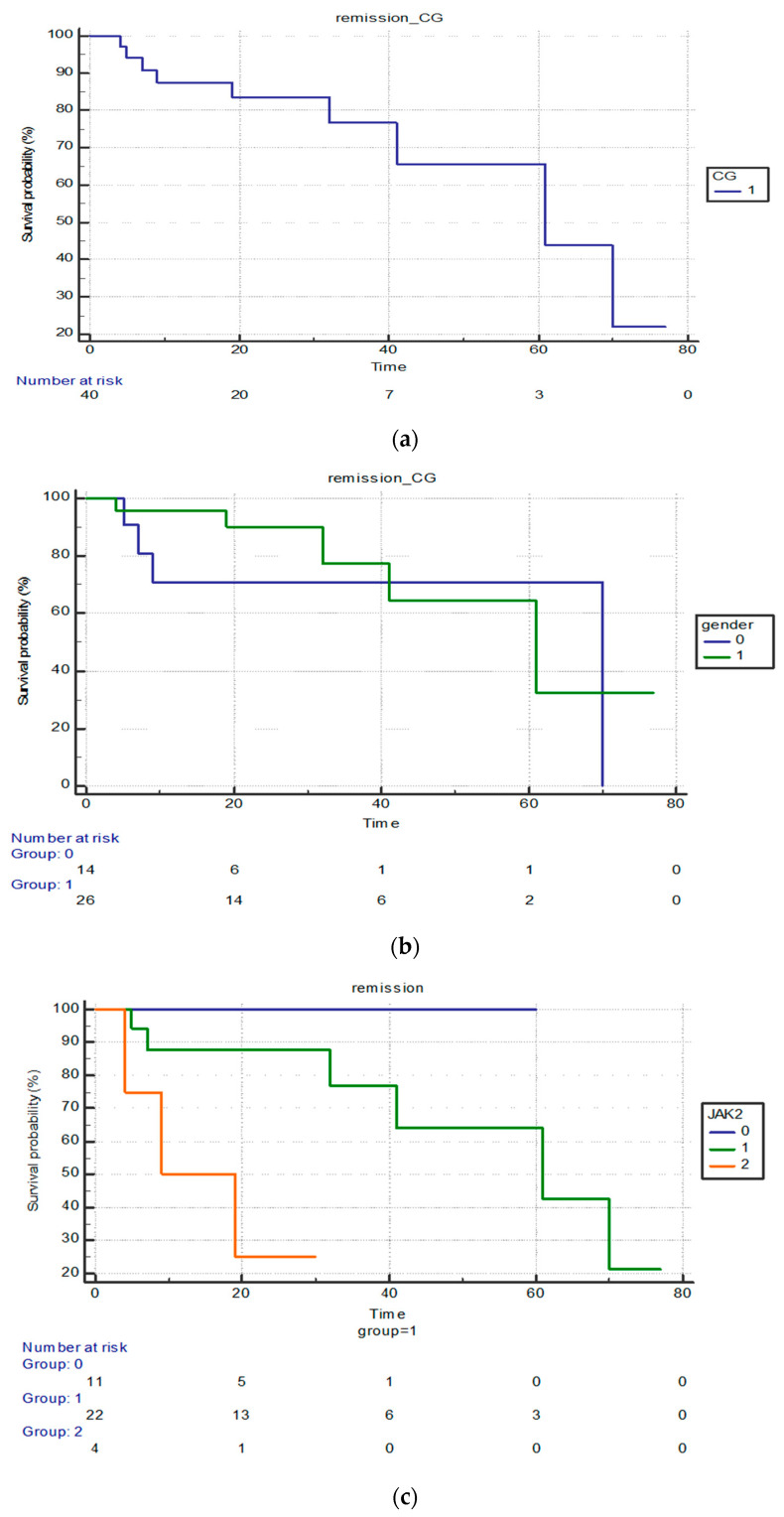
(**a**) Survival curve for control group (CG—control group). (**b**) Survival in female and male subgroups (0—female; 1—male). (**c**). Differences in survival according to JAK status in CG (0—negative JAK2 status; 1—positive homozygous JAK2 status; and 2—positive heterozygous JAK2 status).

To perform the Kaplan–Meier analysis, we excluded three patients from the control group due to an earlier onset of the disease. Figure 1a presents the survival curve for the control group, which shows that the survival period was a maximum of 80 months for nearly all 42 patients included in the analysis, equivalent to 6.6 years of additional life after diagnosis.

We did not find a statistically significant difference between the sexes in the control group in terms of survival (*p* = 0.4372) (Figure 1b). The hazard ratio for survival in males was 56.9% that in females. The average survival for males in the sample was 70 months, compared to 61 months for the female subgroup.

Figure 1c shows the distribution of survival of patients with different JAK2 statuses in the control group (0—negative JAK2 status; 1—positive homozygous JAK2 status; and 2—positive heterozygous JAK2 status). There is a statistically significant difference between the survival between the three groups (*p* = 0.0013).

The hazard ratio shows that, on the population level, the mortality risk is 2.5 higher in the patients with a negative JAK status. The average survival of JAK2-negative patients was 9 months while the heterozygous JAK2 patients survived 19 months on average.

##### Survival in the Group on Ruxolitinib

**Figure 2:**
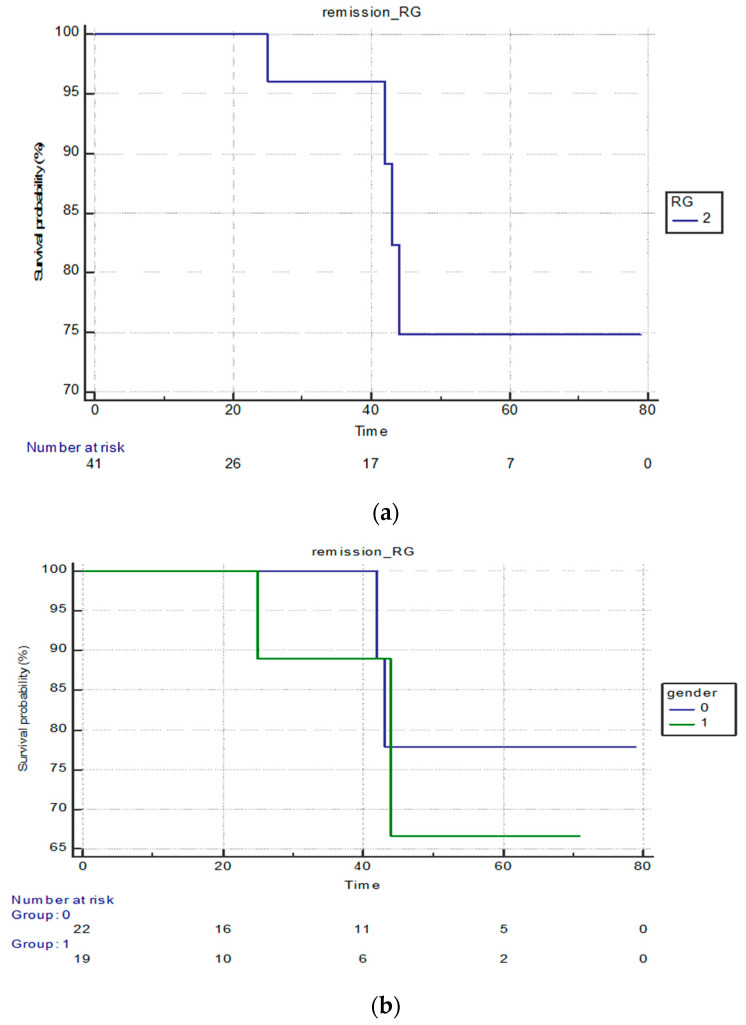
(**a**) Survival in the group on ruxolitinib (RG—ruxolitinib group). (**b**) Survival of females and males in the ruxolitinib group (0—female; 1—male).

In the ruxolitinib group, 75% of patients experienced a >80 month survival, which already shows a vastly improved survival probability compared to the standard therapy group. Additionally, for these 75% on ruxolitinib, their survival was extended by a minimum of 10 months as some patients survived longer than 90 months (Figure 2a).

There was no statistically significant difference between female and male survival in this group (*p* = 0.5789; Figure 2b).

In terms of the JAK2 status, we also found that the survival of the JAK2-negative patients was shorter and this difference was statistically significant (*p* = 0.0065). The hazard ratio for mortality in JAK2-negative patients was 4216 times higher than that for JAK2-positive patients.

We found leukemic transformation in three patients in the active group. We do not have this information for the control group as testing for CALR status is not a routine practice and is only performed for JAK2-negative patients. Out of the nine negative patients in the active group, six has a CALR status. Five patients had a positive CARL status and one had a negative status. This patient was a triple negative patient (for JAK, CALR, and MPN).

##### Comparison of Survival for Both Groups

**Figure 3:**
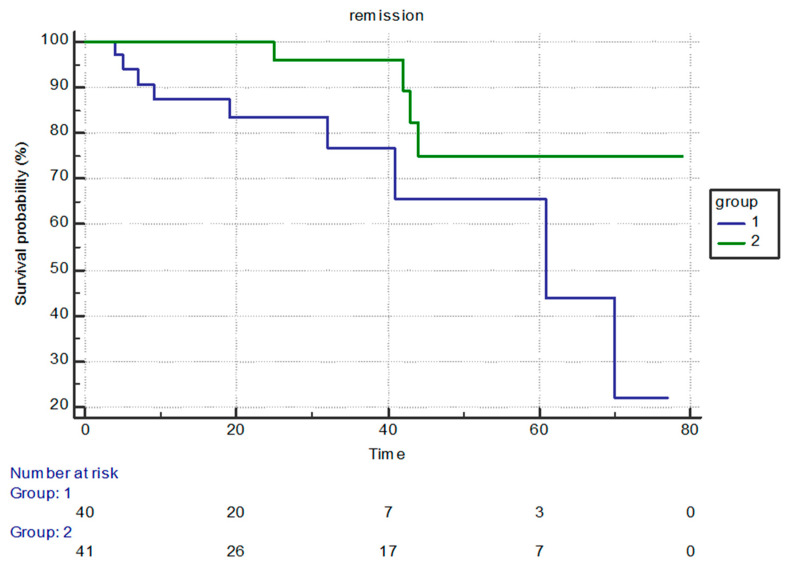
Survival of both groups (1—control group and 2—ruxolitinib group).

Figure 3 shows the Kaplan–Meier curves comparing both the control and active ruxolitinib groups. At the 80th month mark, when all patients on standard therapy had passed away, 75% of patients on ruxolitinib were still alive. This difference was statistically significant (*p* = 0.0161).

The mortality risk in the ruxolitinib group was 24.77% of that in the control group, indicating a 75.33% improved chance of survival. This is a 75.33% reduction in the mortality risk in the sample. Due to the small sample size, the confidence intervals were large, indicating that, on the general population level, mortality risk in the ruxolitinib group varied between 7.9% and 77.18% of the risk in the control group. The minimal risk reduction in the treated group was between 32.98% and 92.1% of the risk in the control group on the population level.

#### 3.2.2. Subgroup Analysis of the Clinical Results in the Ruxolitinib Group

Table 2 presents the data on changes in clinical outcomes over the follow-up period, with clinical examinations conducted at baseline, six months, and twelve months, as well as the statistical analyses of these changes.

The median values of the indicators for each measurement were compared, rather than the arithmetic mean since the median is characteristic of the mean of the observed distribution.

There was a decrease in blood parameters (leukocytes, hemoglobin, platelets), as well as in the spleen size. However, the blood tests remained within the reference intervals. During the first six months, the size of the spleen decreased by 6%, and during the second six months, there was another 9% decrease. A decrease in the MPN SAF values was also observed.

Decreasing spleen sizes and MPN SAF values are indicative of a positive response of patients to ruxolitinib and an improvement in their condition, as well as a reduction in disease burden. All the changes in clinical parameters were statistically significant except changes in lactate dehydrogenase (LDH). This indicates that treatment with ruxolitinib sustainably and significantly improves clinical outcomes.

Similarly, the dependence on transfusions decreased, as well as the severity of symptoms, and these changes were also statistically significant. Thus, we can state that ruxolitinib is an effective therapy for patients with MF, as it improved the patients’ condition and clinical indicators in the one-year treatment period. If these data are added to the patient survival calculations from the previous section, it is evident that through the improvement of clinical parameters and the reduction in disease burden, the life of patients treated with ruxolitinib is also prolonged.

The frequency of adverse events was 22.7% for anemia (reported in 15 people in the group) and 6.1% for thrombocytopenia (reported in 4 people), with no data for 6 people.

From a statistical point of view, it is of interest to track the correlations between patient characteristics, clinical indicators, and disease severity, and to determine their prognostic significance. Several correlation analyses were performed to assess the relationship between treatment outcomes and patient characteristics; the results are shown in Table 3 where the statistically significant correlations (*p* < 0.05) are marked in red.

A negative, weak correlation was found between hemoglobin at the beginning of the study and the change in the spleen size (Table 3). This means that with an increase in the size of the spleen, hemoglobin decreases. Negative relationships were found between JAK status, age, and leukocytes at the beginning of the study. Age was the factor that correlated with the greatest number of patient characteristics. The correlation between age and time since diagnosis of MF (r = −0.364), platelets at baseline (r = −0.398), and hemoglobin at baseline (r = −0.364) were negative which could be interpreted as a higher risk of poor clinical outcomes in older patients and a more negative long-term prognosis. There was a positive correlation between leukocytes at baseline, risk group, and the degree of MF, which is at risk of worsening with age. Similar comments can be made regarding the other correlations between patient characteristics at the start of ruxolitinib therapy.

Table 4 presents the correlations between symptom severity, as measured by the MPN SAF, and the patient characteristics. Unfortunately, no relationships were found between symptom severity and the patient characteristics, as well as between transfusion needs and patient characteristics. The rest of the correlations are the same as those shown in Table 3 since the data are the same.

### 3.3. Prognostic Significance of Changes in Clinical Parameters

From a clinical point of view, it is important to answer the question of whether the statistically significant changes in the clinical parameters possess prognostic value to predict the future development of the disease. This question was investigated by performing a linear regression analysis of changes in the most important clinical parameters.

**Figure 4:**
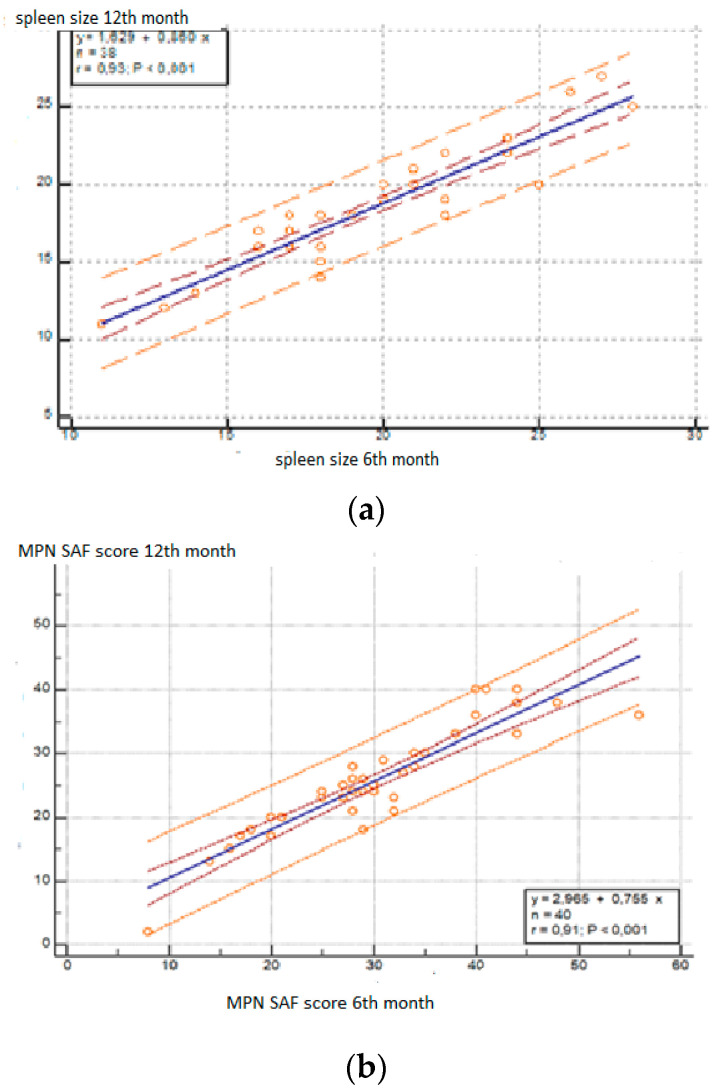
(**a**) Linear regression model of the changes in spleen size during observation period. (**b**) Linear regression model of the changes in symptoms severity during observation period.

The results of the linear regression analysis of the spleen size data at the beginning and after 6 and 12 months show that the analytical model that describes the changes is expressed by the formula: y = 1.6287 + 0.8598x. (Figure 4a) The model adequately describes the investigated dependence (*p* < 0.001) and the independent variable (x) explains 87% of the variation in the dependent variable (y) of the model, i.e., based on spleen size after 6 and 12 months, the size after another 12 months can be predicted. This dependence is linear and did not change over time.

The results of the linear regression analysis of the changes in the severity of symptoms at the beginning and after 6 and 12 months show that the analytical model that describes the changes is expressed by the formula: y = 2.9650 + 0.7551x (Figure 4b). The model adequately describes the investigated dependence (*p* < 0.001) and the independent variable (x) explains 83% of the variation in the dependent variable (y) of the model.

Both linear regression models can be used to predict the mean value of spleen size and MPN SAF score based on the values at the beginning or after 6t and 12 months. With the regression equation and known values of spleen size and MPN SAF score, physicians can predict future changes in these two variables.

## 4. Discussion

Predicting the outlook for MF is difficult and depends on many factors. These factors are used in the international prognosis scoring system (IPSS) to help physicians predict the average number of years of survival. The life expectancy for a person with myelofibrosis may range from 1 to 15 years or more. This can depend on individual risk factors, including age, disease progression, and response to treatment [16]. It is important to note that these survival estimates are based on survival averages and currently available treatments. As newer treatments are developed, survival rates may also change [17]. Patients with one of these risk factors are expected to have an average survival rate of about six years, while patients with three or more risk factors have a lower expected survival rate of around one to three years. In this study, we explored age, JAK status, type of MF, and response to therapy.

In this study, we explored long- and intermediate-term treatment results of MF therapy with ruxolitinib in comparison to standard therapy in a real-life clinical practice. Both analyses confirmed that ruxolitinib increased survival and improved clinical results. These results confirmed the opinion that myelofibrosis management has come a long way in the last 10 years. This is mainly that due to advancements in therapy and in the growing list of effective myelofibrosis drugs. Unfortunately, not all medicines are available in all countries for the benefit of patients [18,19,20,21,22].

Ruxolitinib is the only JAK inhibitor reimbursed in Bulgaria, which is why our analysis focused on this specific therapy [23]. The control group information was obtained retrospectively, which is why there was no standard therapeutic protocol applied for this group, and we could not collect longitudinal observational data for the clinical results, which could be considered a limitation of our study. 

Similar to our study, Schain F. et al. [12] explored survival of Swedish and Norwegian MF patients on ruxolitinib therapy. They retrospectively collected information from the national registries of patients with MF during 2001–2016. The patients were followed from ruxolitinib initiation until death or end of follow-up. The relative survival was found to be 0.80 and 0.52 during the first and fourth year, respectively, after the initiation of ruxolitinib therapy. The difference with our study is that they compared the survival of ruxolitinib patients with a matched general population. A similarity is that they also had a small sample size of 47 and 48 patients in Norway and Sweden, respectively. Although our study was performed in one hospital, it included a similar number of patients with MF (45 vs. 66 on ruxolitinib). This is partly due to the rarity of the disease, as well as the fact that SHATHD is the national reference center for hematological malignancy therapy. The overall number of patients with MF is estimated to be around 280 and we can consider our sample of 66 patients as be representative for the MF population.

In contrast with the follow-up studies of the COMFORT-I and COMFORT-II trials where patients discontinued ruxolitinib within 3 to 5 years (25%), we had a relatively small number of patients for whom the information about the discontinuation was available [24].

The other study exploring the long-term survival of patients on ruxolitinib therapy was based on a pooling analysis of the results from the COMFORT-I and COMFORT-II trials [25]. The authors found that the risk of death was reduced by 30% among ruxolitinib patients compared with patients in the control group who were treated with either a placebo or the best available therapy. In contrast, our study found a 75.33% decrease in mortality risk. This could be explained by the real-life settings in which this study was performed as well as the longer period of observation.

The two RCTs reported a decrease in spleen size of 35% after 12 months while we observed a 15% decrease after 12 months [9,10]. In our study, if the same rate of decrease continued, the spleen size reduction would be close to that manifested in the RCTs are 24 months. The same studies reported an improvement of 50% or more in the MPH TSS score at 24 weeks, while we observed a 63% decrease after 12 months of therapy. Therefore, our results supported the efficacy data for ruxolitinib from the RCTs.

In a real-life study of ruxolitinib [26], the patients had a mean age of 63.5 years, and for 13.1 months, 40% of the patients took the recommended dose. The Kaplan–Meier estimate of the median survival from ruxolitinib initiation was 44.4 months (95% CI, 38.8–50.2 months). In contrast with this study, we only followed patient survival during the period of observation and found out that for almost 7 years, 75% of the patients were still alive and will continue to live for more than 80 months.

Compared with the control group, patients treated with ruxolitinib had improved survival and 75% of these patients will be alive after 80 months. Expectedly, JAK status affected the survival, and in our cohort, JAK-negative patients had a shorter survival [27,28].

We observed decreases in leukocyte, hemoglobin, and platelet concentrations. These changes in blood counts can be related to both the treatment and the disease. We cannot use the blood counts to make conclusions about the treatment results. The blood results are important for treatment decisions like dose modifications or to confirm disease progression [29]. The reduction in the spleen size is one of the primary aims of the treatment with ruxolitinib. In real-life practice, we observed a reduction in the spleen size, as reported in the clinical trials.

The other aim of the treatment with ruxolitinib is the improvement of symptoms and the quality of life. The decrease in the MPN SAF score shows that there was an improvement in the symptoms for patients treated with ruxolitinib [30]. We also observes a reduction in transfusion dependence during the course of treatment. This is another point indicating improved quality of life. We found a correlation between spleen size and MPN SAF score which can be used to predict changes in the two variables.

We reported hematological toxicity as it is described in the SPC. Cases of anemia and thrombocytopenia were reported, and the dose of the drug was modified as recommended; we did not have any cases of treatment termination due to adverse events [31].

The other limitations of our study are that it observed patients in only one hospital but, as it was pointed out earlier, it is the reference center for hematological malignancies and treats patients from all over the country. The clinical data for the active and control groups were missing, as was the classification of patients with MF into the four risk categories, complete information on JAK2 status, and secondary MF cases. Therefore, the small number of patients was further reduced for a complete comparison between the active and control groups, and this is another limitation of the study.

## 5. Conclusions

This study shows that MF patients in real-life clinical practice treated with ruxolitinib have improved clinical signs of the disease and decreased symptom severity, and they survive longer than patients on standard therapy. The improvements were similar to those reported in the clinical trials.

## Figures and Tables

**Table 1:** Demographic characteristics of the patients.

Characteristic	Control Group	Ruxolitinib Group
	Number (% or SD)	Number (% or SD)
Total number	45	66
Female	17 (38%)	31 (47%)
Male	28 (62%)	35 (53%)
Age at diagnosis	From 30 to 76 (average 60.9; SD 8.4)	From 42 to 72 (average 57.1; SD 9.2)
Length of disease	From 1 month to 10 years (average 2.6; SD 2.04)	From 0.5 to 10 years (average 3.3; SD 1.96)
Type of MF		
Primary	40 (89%)	21 (32%)
Secondary	0 (0%)	22 (33%)
No information	5 (11%)	23 (35%)
JAK status		
Positive homozygotes	25 (56%)	15 (23%)
Positive heterozygotes	4 (9%)	18 (27%)
Negative	12 (26%)	9 (14%)
No information	4 (9%)	24 (36%)
On ruxolitinib therapy	1 (2%)	66 (100%)
Number of transfusions	23 (51%)	7 (11%)

**Table 2:** Changes in clinical outcomes over the follow-up period in the active group.

Indicator	Median (25th–75th) 1st Measurement	Median (25th–75th) 2nd Measurement	Median (25th–75th) 3rd Measurement	*p* Value
Leukocytes (g/L) *	12.0 (7.0–23.0)	9.0 (5.0–14.0)	9.0 (5.0–12.0)	<0.00003
Hemoglobin (g/L) *	127.0 (100.0–144.7)	111.0 (94.0–132.0)	107.0 (96.0–125.2)	0.00987
Thrombocytes (g/L) *	319.0 (201.7–446.7)	222.0 (131.7–305.2)	176.0 (125.7–271.2)	<0.00003
Spleen size (mm)	21.5 (19.0–25.0)	18.5 (16.0–22.0)	18.0 (16.0–20.0)	<0.00003
MPN SAF score	39.0 (31.0–48.0)	29.0 (25.0–34.0)	24.5 (20.0–30.0)	<0.00003
Number of transfusions	3.5 (3.0–4.0)	2.5 (2.0–3.0)	2 (2.0–3.0)	0.00558
Ruxolitinib dose *	20.0 (15.0–20.0)	15.0 (15.0–20.0)	14.0 (5.0–20.0)	0.00003

Legend: *p*-values were adjusted using the Bonferroni correction method for multiple comparisons. * g/L (gram/litter).

**Table 3:** Correlation analysis between patient characteristics (only the statistically significant correlations are presented).

		Age	Duration of Treatment	Leucocytes	Risk Group	Thrombocytes—Beginning	Degree of MF	Hemoglobin	Ruxolitinib Dose/mg
Changes in spleen size	Correlation Coefficient							−0.357	
	Significance Level P							0.0236	
	N							40	
JAK2 status	Correlation Coefficient	−0.455		−0.326					
	Significance Level P	0.0025		0.0349					
	N	42		42					
Age	Correlation Coefficient		−0.364	0.344	0.478	−0.398	0.423	−0.364	
	Significance Level P		0.0178	0.0255	0.0014	0.0091	0.0081	0.0177	
	N		42	42	42	42	38	42	
Leukocytes	Correlation Coefficient				0.317	0.331			
	Significance Level P				0.0409	0.0325			
	N				42	42			
Risk group	Correlation Coefficient							−0.381	−0.452
	Significance Level P							0.0129	0.0027
	N							42	42
Thrombocytes	Correlation Coefficient							0.308	
	Significance Level P							0.0472	
	N							42	
Degree of MF	Correlation Coefficient							−0.5	
	Significance Level P							0.0014	
	N							38	

Legend: Correlation coefficient—indicates the degree of correlation; *p*-value shows statistical significance; and N is the number of observations.

**Table 4:** Correlation analysis between the characteristic of patients and symptoms severity (only statistically significant correlations are shown).

		Leucocytes	Risk Group	Thrombocytes	Hemoglobin	Changes in Spleen Size	Age
Changes in MPN SAF	Correlation Coefficient	−0.362					
	Significance Level P	0.0185					
	n	42					
Changes in transfusion needs	Correlation Coefficient		−0.372		0.465		
	Significance Level P		0.0154		0.0019		
	n		42		42		
Leukocytes	Correlation Coefficient		0.317	0.331			0.344
	Significance Level P		0.0409	0.0325			0.0255
	n		42	42			42
Risk group	Correlation Coefficient				−0.381		0.478
	Significance Level P				0.0129		0.0014
	n				42		42
Thrombocytes	Correlation Coefficient				0.308		−0.398
	Significance Level P				0.0472		0.0091
	n				42		42
Hemoglobin— beginning	Correlation Coefficient					−0.357	−0.364
	Significance Level P					0.0236	0.0177
	n					40	42

Legend: Correlation coefficient—indicates the degree of correlation; *p*-value shows statistical significance; and n is the number of observations.

## Data Availability

Data can be obtained from authors upon reasonable request.
